# Bacterial Communities Present Distinct Co-occurrence Networks in Sediment and Water of the Thermokarst Lakes in the Yellow River Source Area

**DOI:** 10.3389/fmicb.2021.716732

**Published:** 2021-10-22

**Authors:** Ze Ren, Cheng Zhang, Xia Li, Kang Ma, Zhe Zhang, Kexin Feng, Baoshan Cui

**Affiliations:** ^1^Advanced Institute of Natural Sciences, Beijing Normal University, Zhuhai, China; ^2^School of Environment, Beijing Normal University, Beijing, China; ^3^School of Engineering Technology, Beijing Normal University, Zhuhai, China; ^4^College of Arts and Sciences, Beijing Normal University, Zhuhai, China

**Keywords:** permafrost, thermokarst lakes, co-occurrence network, modularity, keystone taxa

## Abstract

Thermokarst lakes are a ubiquitous and important landscape feature in cold regions and are changing tremendously due to the accelerated climate change. In thermokarst lakes, sediment and water are two distinct but highly interconnected habitats, harboring different bacterial communities in terms of taxonomic composition. However, the co-occurrence networks of these bacterial communities remain unclear. Here, we investigate the co-occurrence ecological networks of sediment and water bacterial communities for thermokarst lakes in the Yellow River Source Area on the Qinghai-Tibet Plateau. The results show that the bacterial communities construct distinct co-occurrence networks in sediment and water. The metacommunity network was parsed into four major modules formed by the operational taxonomic units (OTUs) enriched in sediment or water independently, and water-enriched OTUs exhibited much closer interconnections than sediment-enriched OTUs. When considering the sediment and water bacterial networks separately, different topological properties and modular patterns present: the sediment bacterial network was more clustered while the modules less responded to the environmental variables. On the contrary, the water bacterial network was more complex with the OTUs more interconnected and its modules more responded to the environmental variables. Moreover, the results of the structural equation model suggest that, by the influence of environmental variations on individual modules, the water bacterial communities would be more vulnerable under the fact of accelerating climate change. This study provides insights beyond a conventional taxonomic perspective, adding our knowledge of the potential mechanisms structuring bacterial community assembly and improving our prediction of the responses of this fast-changing ecosystem to future climate change.

## Introduction

Thermokarst lakes, also known as thaw lakes, are formed as a result of thawing ice-rich permafrost, acting as a ubiquitous landscape feature in the cold region with ecological and biogeochemical importance (Kokelj and Jorgenson, 2013; Chin et al., 2016; In’T Zandt et al., 2020; Manasypov et al., 2021). Thermokarst lakes evolve through vertical and horizontal permafrost degradation with the lake areas ranging from a few square meters to hundreds of square kilometers (Grosse et al., 2013). Permafrost covers about 24% of the Northern Hemisphere and is suffering serious climate change (Qiu and Cheng, 1995; Zhang et al., 1999), especially in the Arctic/sub-Arctic and Qinghai-Tibet Plateau, where the atmosphere has warmed faster than other areas on the Earth (Yao et al., 2000; Mountain, 2015). As a consequence of accelerating permafrost degradation, thermokarst lakes are changing tremendously in size and abundance during the evolution process of formation, expansion, shrinkage, and disappearance, causing substantial impacts on regional and global ecosystem structure and biogeochemical processes (Yoshikawa and Hinzman, 2003; Smith et al., 2005; Lantz and Turner, 2015; Pastick et al., 2019). However, our knowledge of the thermokarst lake ecosystem structure and function is scarce compared with extensively studied temperate lakes.

Bacterial communities exhibit high diversities and variabilities in taxonomic and functional composition and play crucial roles in ecosystem structuring and various biogeochemical processes (Fuhrman, 2009; Miki et al., 2014). Previous studies suggest that bacterial communities are influenced by biogeographic factors differentially in different ecosystems (Martiny et al., 2006). In lake ecosystems, sediment and water are two distinct but highly interconnected habitats (Carter et al., 2003; Parker et al., 2016), which host diverse but substantially different bacterial communities in taxonomic and functional composition (Lozupone and Knight, 2007; Roeske et al., 2012; Ren et al., 2019a, b). Especially in thermokarst lakes, sediment and water have intimate connections during the formation and evolution processes of the lake. Thawing permafrost generates the initial sediment and water from the frozen soil, and the horizontal and vertical permafrost degradation contributes to sediment and water continuously (Kokelj et al., 2005; Vonk et al., 2015; de Jong et al., 2018). Moreover, the accelerated climate change intensified the sediment-water interactions. Therefore, understanding the structuring mechanisms and distribution patterns of bacterial communities in sediment and water is important to promote our understanding of the connections and distinctions between these two habitats.

In natural ecosystems, bacteria taxa often coexist with each other with intense interactions to form complex communities rather than existing alone as individual populations (Fuhrman, 2009; Zhou et al., 2010; Barberan et al., 2012; Shi et al., 2016). These interactions are crucial in community assembly and imply meaningful biological and biochemical relationships between different taxa (Weiss et al., 2016). Therefore, understanding bacterial communities should not be restricted on species-level characteristics, such as taxonomic richness and composition, but also pay attention to interspecific relationships in complex communities. The microbial co-occurrence network is an effective method to understand the assemblage rules of the complex communities (Fath et al., 2007; Deng et al., 2012). For example, this approach can characterize the potential interactions among taxa in a network and compartmentalize the network into sub-clusters of closely associated taxa (Newman, 2006; Fuhrman, 2009; Menezes et al., 2015; Banerjee et al., 2016), integrating high-dimension microbial community data into predicted ecological modules (Menezes et al., 2015; Shi et al., 2016). In many biological systems, modularity (the tendency of a network parsed into modules) is an essential ecological feature to reveal more ecological and evolution properties but is easily overlooked (Parter et al., 2007; Centler et al., 2020). The relationships between bacterial environmental variations and microbial modules as well as between microbial modules and the whole community can improve our understanding of direct and indirect influences of environmental variations on microbial communities (Menezes et al., 2015; Toju et al., 2016; Ren and Gao, 2019). Changes in bacterial networks have important implications for their functioning and vulnerability under future disturbance, such as climate change (de Vries et al., 2018). However, the bacterial co-occurrence network in thermokarst lakes remains an important knowledge gap.

In this study, we aimed to investigate the co-occurrence ecological networks in sediment and water of the fast-changing thermokarst lakes in the Yellow River Source area on the Qinghai-Tibet Plateau. According to the distinct taxonomic composition and beta-diversity pattern of the same bacterial communities (Ren et al., 2021), we hypothesized that bacterial communities construct distinct co-occurrence networks in sediment and water in the thermokarst lakes. The results could provide further insights into the assemblage mechanisms and biogeography patterns of bacterial communities.

## Materials and Methods

### Study Area, Field Sampling, and Chemical Analysis

The field sampling work was conducted in the source area of the Yellow River on the Qinghai Tibetan Plateau in early July 2020 (Supplementary Figure 1). In total, 23 thermokarst lakes were sampled. In every lake, water, and sediment samples were collected. The water samples were collected at a depth of 0.3–0.5 m and filled in three 1 L acid clean bottles. Water samples were transported to and reserved in the laboratory at 4°C for dissolved organic carbon (DOC), total nitrogen (TN), and total phosphorus (TP) analyses. A multiparameter instrument (YSI ProPlus, Yellow Springs, Ohio) was used to measure conductivity and pH of lake water *in situ.* Bacterial samples were collected on a 0.2 μm polycarbonate membrane filter (Whatman, United Kingdom) by filtering 200 mL lake water. Sediment samples were collected using a Ponar Grab sampler. The top 5 cm of the sediment was collected and homogenized. Sediment bacterial samples were connected in a 45 mL sterile centrifuge tube. The remaining sediments were air-dried to determine chemical properties, including pH, conductivity, sediment organic carbon (SOC), TN, and TP. The sediment and water bacterial samples were frozen immediately in liquid nitrogen in the field and stored at −80°C in the lab until DNA extraction. The basic chemical properties of sediment and water samples as well as the taxonomic composition of bacterial communities are summarized in our previous publication (Ren et al., 2021).

### DNA Extraction, PCR, and Sequencing

The sediment and water bacterial samples were used to extract genomic DNA using the DNeasy PowerSoil Kit (QIAGEN, Germany) following the manufacturer’s protocols. The V3-V4 region was amplified using the universal primers: 343F (5′-TACGGRAGGCAGCAG-3′) and 798R (5′-AGGGTATCTAATCCT-3′) (Nossa et al., 2010). An Illumina MiSeq platform (Illumina, San Diego, CA, United States) was used for sequencing the amplicon libraries. The details of DNA extraction, PCR, and purification were described previously (Ren et al., 2021). Raw sequence data were first trimmed by detecting and cutting off the ambiguous bases and low-quality sequences (with an average quality score below 20). Then the paired-end reads (forward and reverse reads) were joined and denoised using QIIME1.9.1 (Caporaso et al., 2010). The effective sequences were clustered to generate operational taxonomic units (OTUs) against the SILVA 132 database (Quast et al., 2013) at a 97% sequence identity level. The sequence data were normalized at a depth of 27,890 sequences per sample to avoid the bias of surveying efforts. Raw sequence data can be accessed at the China National Center for Bioinformation (CRA004269 under the project PRJCA005279).

### Analyses

First, a metacommunity co-occurrence network was constructed based on OTUs presenting in both water and sediment in the same lake for at least eight lakes and had a relative abundance above 0.01% in either sediment or water. In addition, sediment and water bacterial networks were constructed separately based on OTUs that presented in eight or more lakes in the corresponding habitat and had an average relative abundance above 0.01%. For each network, the Spearman correlation was conducted between all pairs of OTUs based on the relative abundance, and the *P-*values were adjusted (*P*_*adjust*_) using adjusted using Benjamini and Hochberg (BH) methods for false discovery (Benjamini and Hochberg, 1995). Only strong (Spearman’s correlation coefficient *R* > 0.80 or *R* < −0.80) and significant (*P*_*adjust*_ < 0.01) correlations were considered to construct the network. To evaluate the significant differences between the constructed network and its corresponding random network, the Erdos–Renyi model was used to generate 999 random networks with the same size (the same number of nodes and edges) as the real network (Erdos and Renyi, 1959). Topological parameters were calculated for real and random networks. The differences of topological parameters between real and random networks were evaluated using the *Z*-test (Zhou et al., 2010; Kuang et al., 2017). Module structures were established, and the modules with more than 10 nodes were recognized as major modules. The within- (Zi) and among-module connectivity (Pi) were calculated for each OTU. According to the within- and among-module connectivity, the topological role of each OTU was identified. The nodes with Zi ≥ 2.5 and Pi < 0.62 were determined as module hubs, which are the highly linked nodes within the modules. The nodes with Pi ≥ 0.62 and Zi < 2.5 were determined as connectors, which are the linking nodes between different modules. The nodes with Zi ≥ 2.5 and Pi ≥ 0.62 were determined as network hubs. Other nodes with Zi < 2.5 and Pi < 0.62 were determined as peripherals. The co-occurrence networks were assessed using the R package igraph 1.2.6 (Csardi, 2013) and visualized using Gephi 0.9.2 (Bastian et al., 2009). A structural equation model (SEM) was conducted to quantify the direct and indirect effects of environmental variables on network modules and the whole bacterial communities in sediment and water using “piecewiseSEM 2.1.2” package (Lefcheck, 2016). All the analyses were carried out in R 4.0.4 (R Core Team, 2017).

## Results

### General Patterns of Metacommunity Network

**FIGURE 1:**
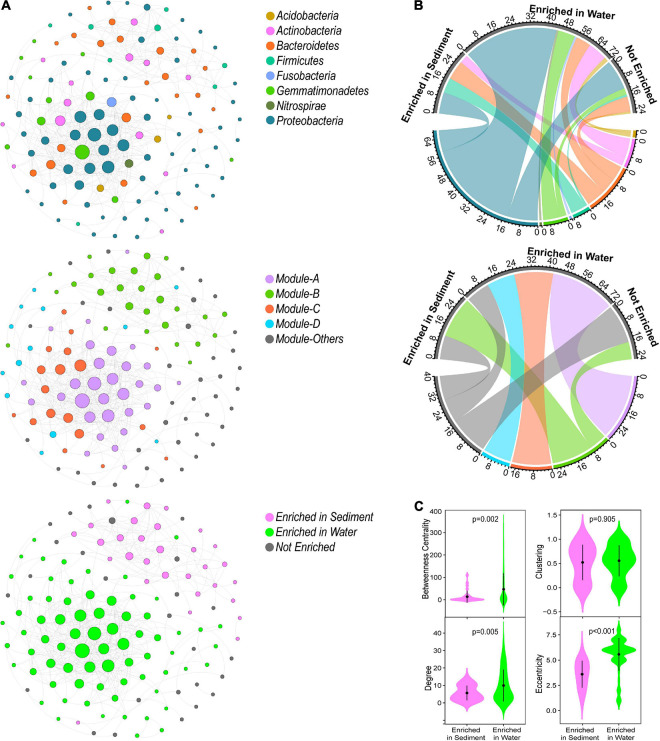
Co-occurrence pattern of metacommunities (a metacommunity was composed of OTUs present in both water and sediment in the same lake) in thermokarst lakes. **(A)** Metacommunity network colored by major phylum, modules as well as enriched habitats. **(B)** Composition of sediment-, water-, and not-enriched OTUs in terms of phylum and modules. **(C)** The node-level topological features of sediment- and water-enriched OTUs. The differences were assessed using Wilcoxon rank-sum test.

The constructed metacommunity co-occurrence network consisted of 471 edges (significant associations) among 128 OTUs (Figure 1 and Table 1). In the metacommunity network, 30 OTUs were enriched in sediment (Figure 1B), belonging to Proteobacteria (*n* = 10), Bacteroidetes (*n* = 9), Firmicutes (*n* = 7), and Actinobacteria (*n* = 4), and 72 OTUs were enriched in water (Figure 1B), belonging to Proteobacteria (*n* = 44), Actinobacteria (*n* = 9), Gemmatimonadetes (*n* = 8), Bacteroidetes (*n* = 7), Acidobacteria (*n* = 2), Fusobacteria (*n* = 1), and Nitrospirae (*n* = 1). Null model analysis indicated that the metacommunity network exhibited significantly higher network diameter, clustering coefficient, and modularity but lower average path length than its random counterpart (the randomly generated network with the same number of nodes and edges) (Table 1), indicating non-random, clustered topology, and modularity structure of the metacommunity network.

**TABLE 1:** Comparison of topological parameters of networks.

	Metacommunity		Sediment		Water	
	Real	Random	Real	Random	Real	Random
Number of nodes	128	128	607	607	1,049	1,049
Number of edges	471	471	2,265	2,265	20,857	20,857
Average degree	7.359	7.359	7.463	7.463	39.765	39.765
Average path length	2.599	2.634 ± 0.012*	5.751^a^	3.413 ± 0.005*	3.518^b^	2.175 ± 0.001*
Diameter	8.000	4.869 ± 0.339*	17.000^a^	6.192 ± 0.396*	11.000^b^	3 ± 0*
Clustering coefficient	0.552	0.058 ± 0.007*	0.416^a^	0.013 ± 0.002*	0.488^b^	0.038 ± 0*
Centralization degree	0.226	0.06 ± 0.012*	0.059^a^	0.016 ± 0.002*	0.153^b^	0.02 ± 0.002*
Centralization betweenness	0.036	0.036 ± 0.009	0.212^a^	0.014 ± 0.002*	0.026^b^	0.001 ± 0*
Modularity	0.446	0.318 ± 0.011*	0.708^a^	0.336 ± 0.004*	0.445^b^	0.126 ± 0.002*

The differences between the real co-occurrence networks and their associated random networks (permutation = 999, values shown mean ± SD) were assessed using a Z-test (*indicates the significant differences of P < 0.05). The differences between sediment and water bacterial network were assessed using a t-test (different lowercase letters indicate the significant difference of P < 0.05).

**FIGURE 2:**
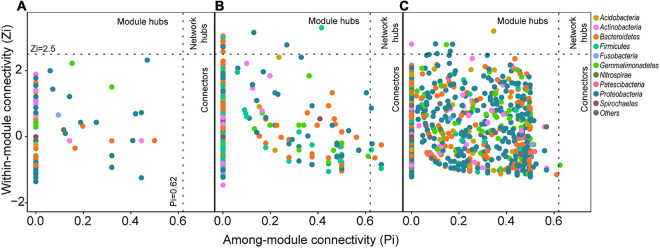
Topological roles of OTUs in the **(A)** metacommunity network, **(B)** sediment bacterial network, and **(C)** water bacterial network. Zi represents within-module connectivity. Pi represents among-module connectivity. Each dot represents an OTU colored by phylum.

In the metacommunity network, bacterial taxa were clustered into four major modules (the module with more than 10 nodes, Figure 1A), which were consisted of OTUs from various phylum groups (Supplementary Figure 2A). However, these modules were formed by the sediment- or water-enriched OTUs independently (Figure 1B). Modules A, C, and D entirely consisted of water-enriched OTUs, and the OTUs in module B were sediment-enriched and non-enriched OTUs (Figure 1B). In addition, we examined the node-level topological features of OTUs enriched in different habitats. Water-enriched OTUs had significantly higher betweenness centrality, degree, and eccentricity than sediment-enriched OTUs (Figure 1C), suggesting that water-enriched OTUs exhibited much closer interconnections than sediment-enriched OTUs. Moreover, according to the topological roles of the nodes, no keystone nodes were found in the metacommunity network (Figure 2A).

### Sediment and Water Exhibited Distinct Co-occurrence Networks

**FIGURE 3:**
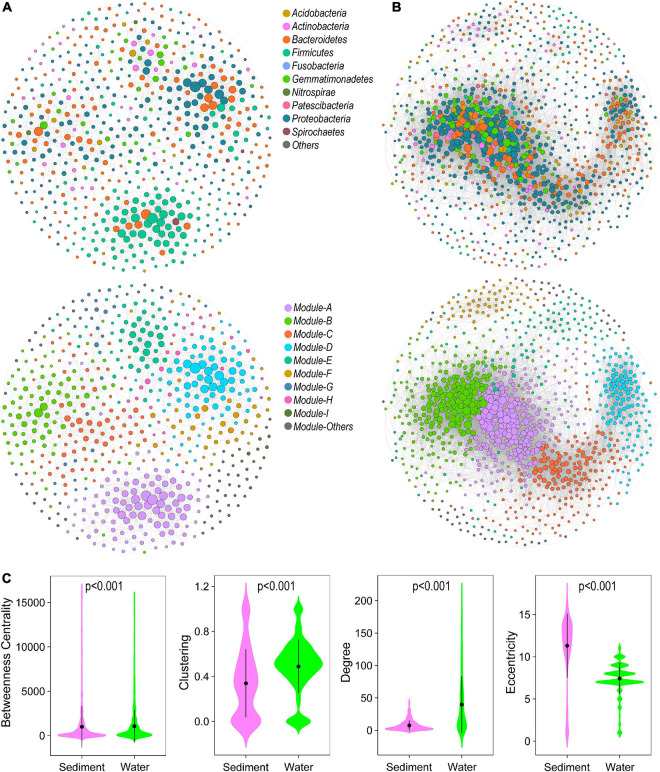
Bacterial co-occurrence network in **(A)** sediment and **(B)** water. The nodes represent the OTUs and were colored by phylum (above) and modules (below). Edges represent Spearman’s correlation relationships. Only strong (Spearman’s *R* > 0.8 or *R* < –0.8) and significant (*P* < 0.05, *P-*values are adjusted using Benjamini and Hochberg methods) correlations are shown. **(C)** The node-level topological features of OTUs in sediment and water bacterial networks. The differences were assessed using Wilcoxon rank-sum test.

In the studied thermokarst lakes, bacterial communities constructed distinct co-occurrence networks in sediment (Figure 3A), and water (Figure 3B). The sediment bacterial network consisted of 2265 associations among 607 OTUs, and the water bacterial network consisted of 20,857 associations among 1049 OTUs (Table 1). Both networks significantly differed from the randomly generated networks with the same number of nodes and edges, suggesting that the real networks were non-random (Table 1). In terms of the network-level topological features, the sediment bacterial network exhibited a higher characteristic path length, diameter, betweenness, and modularity, and the water bacterial network had a higher number of nodes and edges as well as a higher average degree and clustering coefficient (Table 1), indicating that the sediment bacterial network had a more clustered topology (Figure 3A) and the water bacterial network was more complex with the OTUs more interconnected (Figure 3B).

OTUs in different habitats formatted distinct modularity structures. The OTUs in the sediment bacterial network were grouped into nine major modules (the module with more than 10 nodes, Figure 3A). The OTUs in the water bacterial network were grouped into six major modules (Figure 3B). These modules had different taxonomic composition (Supplementary Figure 2). Based on the within-module degree (Zi) and among-module connectivity (Pi) of OTUs in the networks, we identified the keystone nodes (network hubs, module hubs, and connectors) in sediment and water bacterial networks. In the sediment bacterial network, 11 module hubs (five Bacteroidetes OTUs, three Proteobacteria OTUs, two Firmicutes OTUs, and one Gemmatimonadetes OTU) and eight connectors (three Proteobacteria OTUs, two Firmicutes OTUs, two Bacteroidetes OTUs, and one Fibrobacteres OTU) were found (Figure 2B and Supplementary Table 1). In the water bacterial network, only seven module hubs (four Proteobacteria OTUs, one Acidobacteria OTU, one Actinobacteria OTU, and one Gemmatimonadetes OTU) and one connector (Gemmatimonadetes OTU) were found (Figure 2C and Supplementary Table 1). No network hubs were found in both networks. The keystone nodes were not overlapped between sediment and water bacterial networks (Supplementary Figure 3), showing that bacterial species responsible for these important topological roles are different between sediment and water. In addition, we examined the node-level topological features of OTUs in sediment and water bacterial networks. OTUs in the water bacterial network exhibited significantly higher betweenness centrality, clustering, and degree but lower eccentricity than sediment (Figure 2), suggesting that OTUs in the water bacterial network interconnected more closely than OTUs in the sediment bacterial network.

### Linkages Between Bacterial Networks and Environmental Factors

**FIGURE 4:**
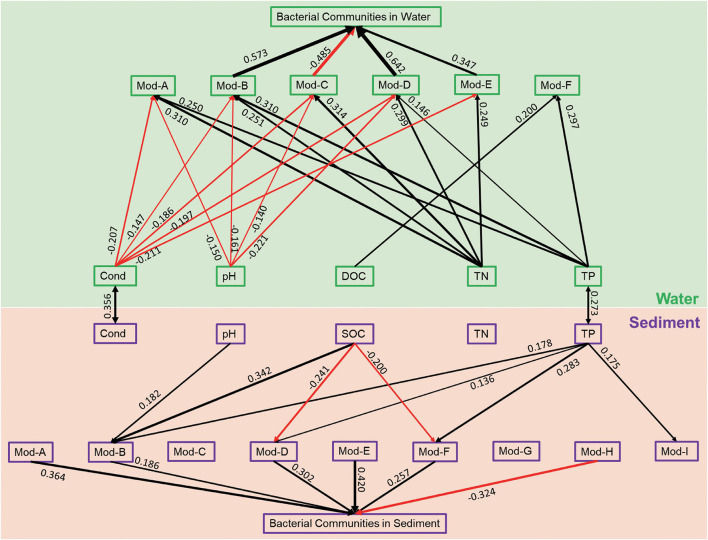
Structural equation model illustrating the relationships between the variation of environmental variables and the beta diversity of network modules and the whole bacterial communities in sediment and water. The solid lines represent significant relationships with red and black arrows represent negative and positive relationships, respectively. The significant path coefficients were shown adjacent to the path.

OTUs in different habitats formatted distinct co-occurrence and modularity structures. SEM was used to reveal the relationships between the variations of bacterial modules and the variations of environmental variables as well as the relationships between the modules and the whole communities. The compositional dissimilarity (β-diversity) for each module and the whole community were estimated using the Bray–Curtis distance based on the relative abundance of OTUs. For the sediment bacterial network, pH only had a significantly positive relationship with module B (Figure 4). SOC had a significantly positive relationship with module B but negative relationships with modules D and F. TP had significantly positive relationships with modules B, D, F, and I. Moreover, the β-diversity of modules A, B, D, E, and F was significantly positive while module H had significantly negative contributions to the β-diversity of the whole bacterial communities in sediment (Figure 4). For the water bacterial network, conductivity had negative, but TN had positive relationships with modules A, B, C, D, and E (Figure 4). pH had negative relationships with modules A, B, C, and D. DOC only significantly correlated with module-F. TP had significant relationships with modules A, B, D, and F. Moreover, the β-diversity of modules B, D, and E had significantly positive while module C had significantly negative contributions to the β-diversity of the whole bacterial communities in water (Figure 4). SEM results suggest that the modules responded differently to the environmental variables. Water bacterial modules had more significant relationships with environmental variables than sediment bacterial modules. Most of the modules contributed positively to the whole communities.

## Discussion

Sediment and water are two distinct but highly linked habitats in lake ecosystems (Parker et al., 2016). In these two habitats, bacterial communities consist of different taxonomic groups (Ren et al., 2019b, 2021), assemble in different rules (Ren et al., 2021), and construct distinct co-occurrence networks. Bacterial communities are typically composed of various taxonomic taxa with potential strong interactions, resulting in complex assemblages. Co-occurrence network analysis is an effective method to offer new insights into assembly mechanisms of complex communities by identifying potential biotic interactions beyond taxonomic structure (Fuhrman, 2009; Faust and Raes, 2012; Ren and Gao, 2019). We first assessed the metacommunity network that was constructed by the taxa presented in both sediment and water in the same lake. As a result, the metacommunity network consisted of 128 OTUs and 471 edges with a clustered topology and modularity structure. This metacommunity network was further parsed into four major modules that were formed by the OTUs enriched in sediment or water. The results suggested that the assembly of the metacommunities was non-randomly determined by habitat preferences, which also resulted in the distinct co-occurrence patterns in different habitats.

Because the sediment and water harbored distinct bacterial communities with a large proportion of taxa only presented in one of the habitats (Ren et al., 2021), we thus constructed the sediment and water bacterial networks separately based on the bacterial taxa living in these two habitats. As a result, we found that the sediment and water bacterial networks differed significantly in topological properties (Figure 3 and Table 1), which can offer more structure information of the co-occurrence network. For example, the clustering coefficient is a measure of the local connectivity and associates with the robustness of a network, the average path length describes node distribution using the average of the shortest path lengths connecting each node to all others, the average degree explains complex pairwise connection using the average number of neighbors for all nodes, and the modularity describes the tendency of the network to contain modules (Newman, 2006). In our study, the bacterial co-occurrence network in water was more complex and more interconnected than that in sediment supported by the network-level properties that the water bacterial network had a higher number of nodes and edges, higher average degree and clustering coefficient as well as lower average path length (Table 1). In the thermokarst lakes, water had a much higher bacterial biodiversity than sediment (Ren et al., 2021). Thus, it is expected that bacterial taxa in water would interact with more taxa, leading to high biotic interactions among taxa and resulting in a complex co-occurrence network. In addition, a higher bacterial diversity could lead to a higher degree of functional redundancy. Microbial species tend to co-occur with each other due to functional capacity and preference (Levy and Borenstein, 2013). High bacterial diversity and functional redundancy provide more probabilities for bacterial taxa to establish relationships with neighborhoods (Tu et al., 2020). Water bacterial taxa also had higher niche width than sediment bacteria (Ren et al., 2021), indicating strong competition among taxa but also similar environmental preferences and lifestyles. Species tend to co-occur more frequently with their competitors in a given environment (Levy and Borenstein, 2013) or with those driven by similar environmental factors (Liu et al., 2019), resulting in complex interactions among taxa. Moreover, these water bacterial communities had a lower β-diversity and lower contribution of turnover component to Sorensen dissimilarity (lower β_turn_/β_sor_ ratio) than sediment bacterial communities (Ren et al., 2021), indicating that bacterial taxa in water had more opportunities to co-occur with other taxa due to low environmental filtering and geographical isolation. However, high connectivity and complexity but lower modularity suggest lower stability of the ecological networks under disturbance (McCann, 2000; Kara et al., 2013; de Vries et al., 2018). Thus, the results also suggest that the water bacterial network is more vulnerable than the sediment bacterial network under accelerated climate change.

For most large, complex systems, modularity is a characteristic of the tendency to contain sub-clusters of members in a network (Barabasi and Oltvai, 2004; Newman, 2006). Module structure can provide more ecological information on complex communities than from a perspective of a taxonomic group (Thompson, 2005), such as synergistic and competitive interactions as well as niche differentiation (Olesen et al., 2007; Freedman and Zak, 2015). First, the metacommunity network was parsed into four major modules, which consisted of OTUs that prefer to present in a certain habitat (enriched in sediment or in water). Habitat preference provides species high interconnections with species preferred to the same habitat. These functionally complementary species form a module finally (Newman, 2006; Freedman and Zak, 2015). Thus, it can be expected that the modules are distinct in many aspects, such as taxonomic composition and environmental responses. In our study, distinct modular structures were also found between sediment and water bacterial networks, which had high modularity with 10 and 6 major modules (modules with more than 10 nodes), respectively. High modularity is usually the result of habitat heterogeneity and high niche diversity across broad spatial scales (Barberan et al., 2012; Ren et al., 2017). The sediment bacterial network had significantly higher modularity than the water bacterial network, suggesting high habitat heterogeneity and further supporting our previous study that sediment bacterial communities had a higher contribution of the turnover component to the Sorensen dissimilarity (higher β_turn_/β_sor_ ratio) due to strong environmental filtering and geographical isolation (Ren et al., 2021). Moreover, the relationships between microbial community modules and environmental variables can improve our understanding of the influences of environmental variation on microbial community assembly (Menezes et al., 2015; Toju et al., 2016). Some modules did not respond, and some modules responded differently to environmental variables. In addition, the modules in the water bacterial network had more significant relationships with environmental variables, further suggesting strong environmental influences on structuring water bacterial communities (Ren et al., 2021). SEM results showed strong relationships between modules and the whole bacterial communities, additionally implying that the vulnerability of water bacterial communities might be indirectly caused by environmental influences on individual modules.

Keystone nodes were also dramatically different between bacterial networks in sediment and water (Supplementary Figure 3). Sediment and water bacterial networks had 11 and 7 module hubs as well as 8 and 1 connectors, respectively. These keystone taxa were important in maintaining network structure and ecosystem stability (Guimerà and Nunes Amaral, 2005). However, due to a lack of taxonomic information at the species or genus level (Supplementary Table 1), it is unattainable to convincingly interpret the potential functions of these keystone taxa. Even so, the limited taxonomic information suggest that most of the keystone taxa (e.g., *Ruminococcaceae*, *Muribaculaceae*, *Bacteroidales_SB-5*, and *Halieaceae*) in the sediment bacterial network play important roles in organic matter degradation, such as lignocellulose degradation (Ren et al., 2019c), carbohydrate degradation (Lagkouvardos et al., 2019), benzene mineralization (Schoch et al., 2020), and alkenes oxidation (Suzuki et al., 2019). In the water bacterial network, the keystone taxa, such as *Microbacteriaceae*, *Rhodobacteraceae*, and *Sphingomonadaceae*, are mainly aerobic and photoheterotrophic or chemoheterotrophic (Kang et al., 2012; Simon et al., 2017). The inferred potential functions of the keystone taxa are consistent with their environmental characteristics that the thermokarst lakes are formed by permafrost thaw, and the sediment is originally permafrost soil with substantial organic carbon and vegetation detritus, which can be released to the up-layer water. The disappearance of these keystone taxa might result in module disintegration or network fragmentation (Widder et al., 2014; Banerjee et al., 2018).

## Conclusion

Ecological network analysis implies a profound and unique understanding of highly complex bacterial communities with insights into community assembly rules and potential taxon interactions. Despite the distinct taxonomic composition and assembly mechanisms of sediment and water bacterial communities in our previous study on these thermokarst lakes, the current study suggests that bacterial communities show distinct co-occurrence patterns in sediment and water. The major modules in the metacommunity network were formed by the OTUs enriched in sediment or water independently. In the metacommunity network, water-enriched OTUs exhibited much closer interconnections than sediment-enriched OTUs. When considering the sediment and water bacterial networks separately, these two networks presented different topological properties and modular patterns. In sediment bacterial network, OTUs were more clustered, and the modules responded less to the environmental variables. In the water bacterial network, however, OTUs were more interconnected, and the modules responded more to the environmental variables. The results indicate that the water bacterial communities were more vulnerable under the fact of accelerating climate change by the influence of environmental variations on individual modules. This study adds to our understanding of the potential mechanisms structuring bacterial community assembly and promotes our predictions of the responses of this fast-changing ecosystem to future climate change.

## Data Availability Statement

The datasets presented in this study can be found in online repositories. The names of the repository/repositories and accession number(s) can be found below: https://bigd.big.ac.cn/gsa/browse/CRA004269, CRA004269, PRJCA005279.

## Author Contributions

ZR designed the study, conducted the fieldwork, did the analyses, and wrote the manuscript. CZ conducted the lab analyses and prepared the manuscript. XL and BC prepared the manuscript. KM conducted the lab analyses. ZZ and KF collected the basic information. All authors contributed to the article and approved the submitted version.

## Conflict of Interest

The authors declare that the research was conducted in the absence of any commercial or financial relationships that could be construed as a potential conflict of interest.

## Publisher’s Note

All claims expressed in this article are solely those of the authors and do not necessarily represent those of their affiliated organizations, or those of the publisher, the editors and the reviewers. Any product that may be evaluated in this article, or claim that may be made by its manufacturer, is not guaranteed or endorsed by the publisher.
