# Supplementary material for: Bacterial Communities Present Distinct Co-occurrence Networks in Sediment and Water of the Thermokarst Lakes in the Yellow River Source Area
Source: Front Microbiol. 2021 Oct 22;12:716732. doi: 10.3389/fmicb.2021.716732 (PMC8569892; doi:10.3389/fmicb.2021.716732)
Supplement: Supplementary file 1 [file Data_Sheet_1.docx]

# Title: Bacterial communities present distinct co-occurrence networks in sediment and water of the thermokarst lakes in the Yellow River Source Area

# Running Title: Bacterial networks in thermokarst lakes

Ze Ren^1,2†^, Cheng Zhang^1,3†^, Xia Li^1,2*^, Kang Ma^2^, Zhe Zhang^4^, Kexin Feng^4^, Baoshan Cui^1,2*^

1 Advanced Institute of Natural Sciences, Beijing Normal University, Zhuhai 519087, China

2 School of Environment, Beijing Normal University, Beijing 100875, China

3 School of Engineering Technology, Beijing Normal University, Zhuhai 519087, China

4 College of Arts and Sciences, Beijing Normal University, Zhuhai 519087, China

**†Authors contributed equally**

***Corresponding Author**:

Xia Li, [lixiabnu@bnu.edu.cn](http://lixiabnu@bnu.edu.cn), Advanced Institute of Natural Sciences, Beijing Normal University, Zhuhai 519087, China

Baoshan Cui, [cuibs@bnu.edu.cn](mailto:cuibs@bnu.edu.cn), School of Environment, Beijing Normal University, Beijing 100875, China

## Supplementary Information


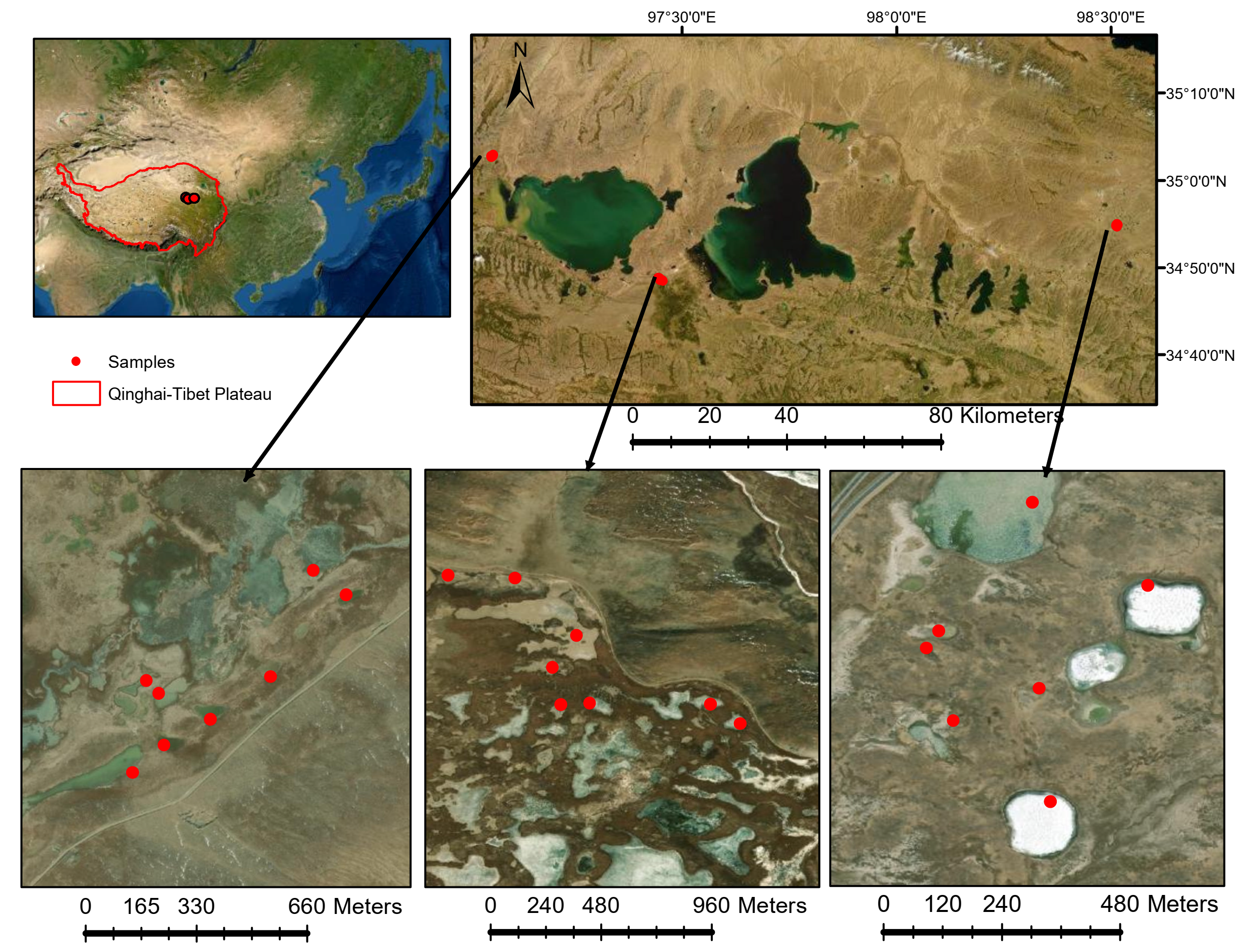


Figure S1 Water and sediment samples were collected from 23 lakes in early July 2020 in the Yellow River Source area on the Qinghai-Tibet Plateau. The map was cited from Ren et al., 2021.


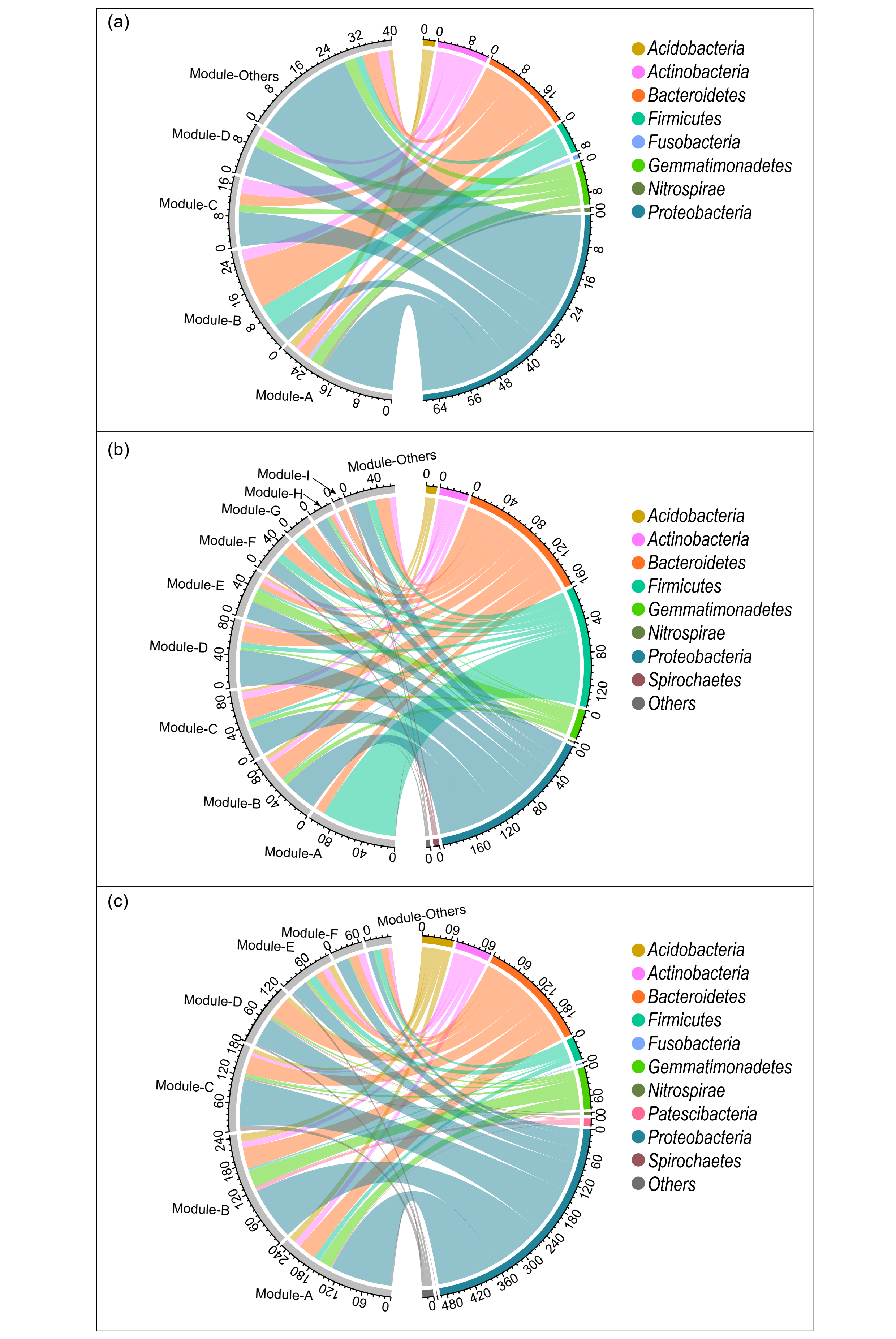


Figure S2 Taxonomic composition network modules shown in number of OTUs. (a) Metacommunity network. (b) Sediment bacterial network. (c) Water bacterial network.


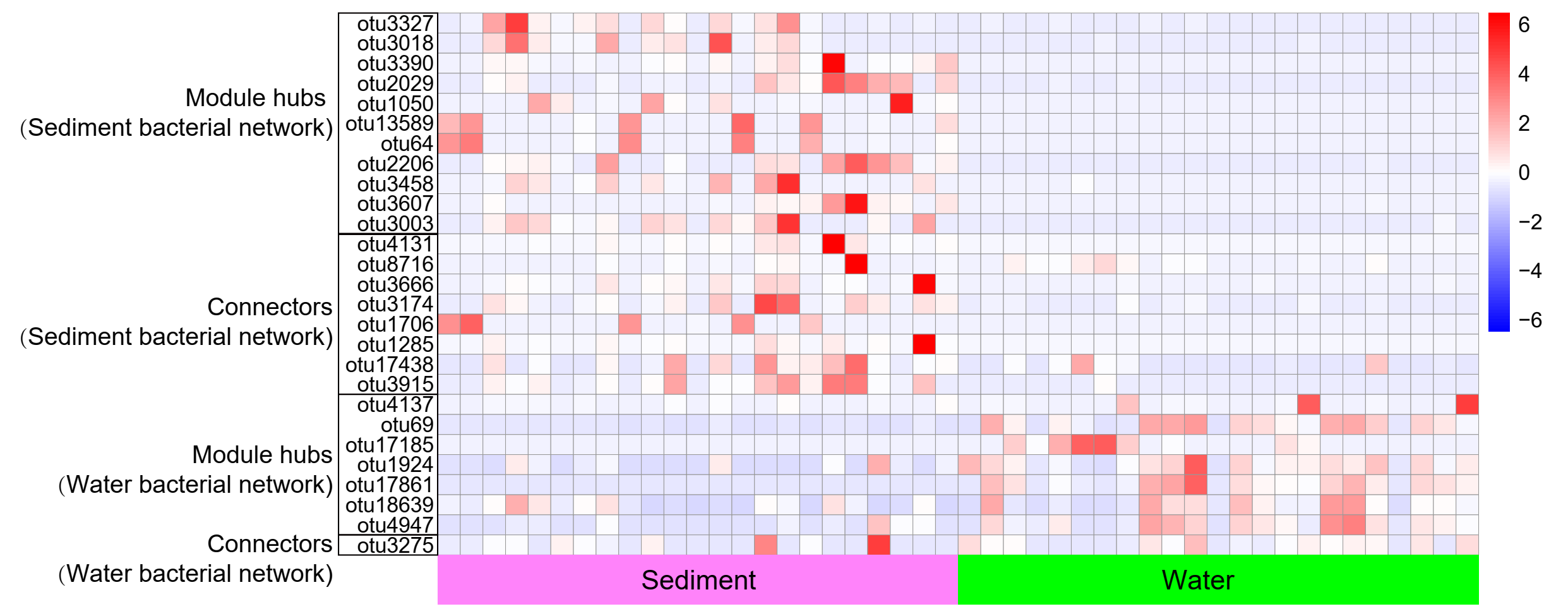


Figure S3 Heat map shown the relative abundance of the keysonte taxa in sediment and water bacterial networks. The relative abundance was normalized using Z-score.

Table S1 List of module hubs and connectors in co-occurrence networks according to the connectivity of each node.

| **OTU ID** | **Pi** | **Zi** | **Category** | **Taxonomy** |
| --- | --- | --- | --- | --- |
| **In sediment bacterial network** | | | | |
| otu1050 | 0 | 3.048 | Module hubs | p_Bacteroidetes; c_Bacteroidia; o_Bacteroidales; f_Muribaculaceae |
| otu64 | 0 | 2.887 | Module hubs | p_Bacteroidetes; c_Bacteroidia; o_Bacteroidales; f_Muribaculaceae |
| otu3003 | 0 | 2.576 | Module hubs | p_Bacteroidetes; c_Bacteroidia; o_Bacteroidales; f_SB-5 |
| otu3458 | 0 | 2.776 | Module hubs | p_Bacteroidetes; c_Bacteroidia; o_Cytophagales; f_Microscillaceae; g_OLB12 |
| otu3327 | 0 | 5.566 | Module hubs | p_Bacteroidetes; c_Bacteroidia; o_Sphingobacteriales; f_Lentimicrobiaceae |
| otu3390 | 0.415 | 3.301 | Module hubs | p_Firmicutes; c_Clostridia; o_Clostridiales; f_Ruminococcaceae; g_Ruminiclostridium |
| otu13589 | 0 | 2.991 | Module hubs | p_Firmicutes; c_Clostridia; o_Clostridiales; f_Ruminococcaceae; g_Ruminococcaceae_UCG-005 |
| otu3018 | 0 | 4.171 | Module hubs | p_Gemmatimonadetes; c_S0134_terrestrial_group |
| otu2206 | 0.269 | 2.782 | Module hubs | p_Proteobacteria; c_Deltaproteobacteria; o_Desulfobacterales; f_Desulfobulbaceae |
| otu3607 | 0 | 2.724 | Module hubs | p_Proteobacteria; c_Gammaproteobacteria; o_Betaproteobacteriales; f_Rhodocyclaceae; g_Azoarcus |
| otu2029 | 0.13 | 3.156 | Module hubs | p_Proteobacteria; c_Gammaproteobacteria; o_Cellvibrionales; f_Halieaceae |
| otu1285 | 0.64 | -0.641 | Connector | p_Bacteroidetes; c_Bacteroidia; o_Bacteroidales; f_Prolixibacteraceae; g_WCHB1-32 |
| otu3174 | 0.667 | -0.246 | Connector | p_Bacteroidetes; c_Bacteroidia; o_Cytophagales; f_Cyclobacteriaceae |
| otu4131 | 0.625 | 0.866 | Connector | p_Fibrobacteres; c_Fibrobacteria; o_Fibrobacterales |
| otu3915 | 0.667 | -0.961 | Connector | p_Firmicutes; c_Clostridia; o_Clostridiales; f_Family_XII; g_Acidaminobacter |
| otu1706 | 0.625 | -0.399 | Connector | p_Firmicutes; c_Clostridia; o_Clostridiales; f_Lachnospiraceae |
| otu8716 | 0.625 | 0.866 | Connector | p_Proteobacteria; c_Alphaproteobacteria; o_Sphingomonadales; f_Sphingomonadaceae; g_Sandarakinorhabdus |
| otu17438 | 0.667 | -0.843 | Connector | p_Proteobacteria; c_Gammaproteobacteria; o_Betaproteobacteriales; f_Burkholderiaceae |
| otu3666 | 0.628 | -0.214 | Connector | p_Proteobacteria; c_Gammaproteobacteria; o_Betaproteobacteriales; f_Rhodocyclaceae |
| **In water bacterial network** | | | | |
| otu4137 | 0.346 | 3.199 | Module hubs | p_Acidobacteria; c_Acidobacteriia; o_Subgroup_2 |
| otu17185 | 0 | 2.804 | Module hubs | p_Actinobacteria; c_Actinobacteria; o_Micrococcales; f_Microbacteriaceae; g_Candidatus_Aquiluna |
| otu17861 | 0.146 | 2.787 | Module hubs | p_Gemmatimonadetes; c_Gemmatimonadetes; o_Gemmatimonadales; f_Gemmatimonadaceae |
| otu1924 | 0.172 | 2.8 | Module hubs | p_Proteobacteria; c_Alphaproteobacteria; o_Rhodobacterales; f_Rhodobacteraceae |
| otu18639 | 0.163 | 2.59 | Module hubs | p_Proteobacteria; c_Alphaproteobacteria; o_Sphingomonadales; f_Sphingomonadaceae; g_Sphingomonas |
| otu4947 | 0.107 | 2.517 | Module hubs | p_Proteobacteria; c_Deltaproteobacteria |
| otu69 | 0.101 | 2.811 | Module hubs | p_Proteobacteria; c_Gammaproteobacteria; o_Betaproteobacteriales; f_Nitrosomonadaceae; g_MND1 |
| otu3275 | 0.625 | -0.861 | Connector | p_Gemmatimonadetes; c_Gemmatimonadetes; o_Gemmatimonadales; f_Gemmatimonadaceae |
